# Hypoxic exercise as an effective nonpharmacological therapeutic intervention

**DOI:** 10.1038/s12276-020-0400-6

**Published:** 2020-03-16

**Authors:** Franck Brocherie, Grégoire P. Millet

**Affiliations:** 1 Laboratory Sport, Expertise and Performance (EA 7370), French Institute of Sport, 11, Avenue du Tremblay, 75012 Paris, France; 2 Institute of Sport Sciences, University of Lausanne, Lausanne, Switzerland

**Keywords:** Biological therapy, Energy metabolism

Dear Editor,

Lee et al.^1^ effectively reported how hypoxia signaling acts differently in organ systems, with protective hypoxia-inducible factor (HIF) stabilization and downstream target pathway activation during acute hypoxic stress and HIF stabilization-related disease pathogenesis in chronic hypoxic conditions. The authors present different ways to target HIF pathways for organ protection, mainly through pharmacologic interventions (activators or inhibitors) or remote ischemic preconditioning/postconditioning (Table 2)^1^.

This review article, published in June 2019, suggests therapeutic applications of hypoxia that were also mentioned on 7 October, 2019, by the Nobel Assembly that delivered the 2019 Nobel Prize in Physiology or Medicine to William Kaelin Jr., Sir Peter Ratcliffe, and Gregg Semenza as follows: “Pharmacologically increased HIF function may aid in the treatment of a wide range of diseases, as HIF has been shown to be essential for phenomena as diverse as immune function, cartilage formation, and wound healing. Conversely, inhibition of HIF function could also have many applications: increased levels of HIF are seen in many cancers as well as in some cardiovascular diseases, including stroke, heart attack, and pulmonary hypertension”. These researchers identified the transcription factor HIF-1α, which determines all oxygen-sensing responses, and clarified that it was regulated by Von Hippel–Lindau tumor suppressor depending on the oxygen level in cells. HIFs regulate more than 300 target genes (e.g., those for vascularization and angiogenesis, inflammation and immune function, autophagy and apoptosis, and redox homeostasis).

In this light, exercising in hypoxic conditions could also be a promising therapeutic approach for targeting HIF signaling in some chronic diseases^2^. Accordingly, there are a growing number of studies demonstrating the therapeutic benefits of combining exercise with hypoxia for blood pressure regulation^3^, chronic obstructive pulmonary disease^4^, obesity^5^, coronary artery disease^6^, or other pathologies. For instance, Gorgens et al.^7^ showed that combining muscle contraction and hypoxia resulted in HIF-1α stabilization and improvements in glucose metabolism and insulin action in human skeletal muscle. With patients demanding to be exposed to safe hypoxia (1800–3000 m), the addition of exercise permits an increase in the overall hypoxia-induced metabolic stress (i.e., greater hypoxemia induced by muscle deoxygenation and systemic desaturation), resulting in putative physiological/therapeutic responses that are not activated to the same extent in a normoxic setting^8,9^. Evidence also suggests that adding high-intensity rather than moderate-intensity exercise to hypoxic stimuli triggers greater adaptations (e.g., improvements in muscle oxygen homeostasis and tissue perfusion induced by enhanced mitochondrial efficiency, control of mitochondrial respiration, angiogenesis, and the capillary-to-fiber ratio)^10^.

Overall, their findings opened doors for new methods based on any exercise modality that aims to reduce the oxygen supply and increase peripheral deoxygenation superimposed on hypoxia^8,9^. This approach may be relevant in the above-listed pathologies. Therefore, as part of the target and niches mentioned by Lee et al.^1^, exercising in hypoxia, particularly at high intensity, might be a valuable and viable nonpharmacological therapeutic strategy. Further investigation is warranted to identify optimal and individually tailored hypoxic exercise regimens and their synergistic effects with (accompanying) medication.
